# Plate osteosynthesis of fractures of the shaft of the humerus: comparison of limited contact dynamic compression plates and locking compression plates

**DOI:** 10.1007/s10195-014-0290-2

**Published:** 2014-04-01

**Authors:** Ashutosh Kumar Singh, Nidhi Narsaria, R. R. Seth, S. Garg

**Affiliations:** 1 Department of Orthopedics, Mayo Institute of Medical Sciences, C 1/157, Vishesh Khand, Gomti Nagar, Barabanki, 226010 Uttar Pradesh India; 2 Mayo Institute of Medical Sciences, Barabanki, India

**Keywords:** Limited contact dynamic compression plate, Locking compression plate, Fracture shaft of humerus, Dynamic compression

## Abstract

**Background:**

The aim of this retrospective study was to compare outcomes and complications of displaced fractures of the shaft of the humerus treated with limited-contact dynamic compression plates (LCDCPs) and locking compression plates (LCPs).

**Materials and methods:**

Two hundred and twelve patients with displaced fractures of the shaft of the humerus, treated with plate osteosynthesis from January 2005 to December 2009 were reviewed. One hundred and two patients (group A) were treated with LCDCP osteosynthesis and 110 patients (group B) were treated with LCP osteosynthesis. Clinical and radiological assessments were made at monthly intervals for the first 6 months and then at 2-month intervals for the next 6 months. Primary outcome measures like operative time, duration of hospital stay, time to fracture union, union rate and secondary outcome measures (functional outcome and complications such as infection, malunion, delayed union, nonunion, implant failure and iatrogenic radial nerve palsy) were compared between both groups. The ULCA scoring system and Mayo elbow performance index (MEPI) were used to assess shoulder and elbow functions, respectively. Rodriguez-Merchan criteria were used to assess the functional outcomes of the fracture fixation.

**Results:**

There was no significant difference found between the two groups in terms of primary outcome measures. According to Rodriguez-Merchan criteria, comparison of functional outcomes of both groups showed insignificant difference (*p* = 0.48). There was no significant difference found between the two groups regarding mean ULCA score (*p* = 0.34) and mean MEPI sore (*p* = 0.54). In terms of complications, no significant difference was found between the two groups.

**Conclusion:**

This study concludes that the principle of fracture fixation was more important than plate selection in fractures of the shaft of the humerus.

**Level of evidence:**

Level 3.

## Introduction

Fractures of the humeral shaft are relatively common, representing between 3 and 5 % of all fractures [1, 2]. Open reduction and internal fixation (ORIF) with plating is generally accepted as the best method of treatment for displaced diaphyseal fractures of the humerus in the adult, with advantages of stable fixation, direct visualization, protection of the radial nerve, and sparing of the adjacent shoulder and elbow joint from injury. Fixation techniques based on compression principles have a lower incidence of nonunion and are found to hasten rehabilitation, with less joint stiffness [3]. Limited-contact dynamic compression plates (LCDCPs), based on principles of dynamic compression and reduced bone-plate contact are used commonly nowadays for operative fixation of fractures of the humeral shaft. Another implant, the locked compression plate (LCP), which has features of compression and point bone-plate contact (minimum contact) is also used for fixation of humeral shaft fractures. Many authors have proved the superiority of locking plates over dynamic compression plates in various cadaveric long-bone models [4–6]. Some biomechanical studies have suggested that locking-plate constructs are stiff and suppress interfragmentary motion to a level that may be insufficient to reliably promote secondary fracture-healing [7–9].

There are very few clinical studies in the literature comparing locked plate and limited-contact dynamic compression plate fixation of humerus shaft fractures. The aim of this study is to investigate whether a difference in plate design improves the outcome in managing a particular chosen group of humeral shaft fractures. We hereby present a retrospective study of humerus shaft fractures treated with ORIF with LCDCP or LCP.

## Materials and methods

During the period of 5 years from January 2005 to December 2009, 280 patients with displaced fractures of the shaft of the humerus were admitted to our hospital for internal fixation. Medical records and X-ray films were retrieved for all of the patients (212 patients) who had undergone open reduction and plate osteosynthesis of the fractured humerus shaft.

The inclusion criteria were as follows:

- Age >16 and <65 years
- Closed diaphyseal fracture of humerus treated with ORIF with either LCDCP or LCP. (Indications for plate osteosynthesis were closed diaphyseal fracture of the humerus with shortening >3 cm, rotation >30°, angulation >20° and conservative treatment failure with loss of reduction).
- Medical records for a follow-up period of at least 1 year should be available for each case included in this study.

Two hundred and twelve patients with unilateral isolated displaced fracture of the shaft of the humerus were included in this study as per the inclusion criteria. The fractures were classified according to the AO alpha-numeric classification system. Patients were excluded if they had open fractures, extra-articular fractures in the proximal and distal 5 cm of the humerus, pathological fractures, incompetent neurological and vascular status of the affected extremity, or any other associated ipsilateral or contralateral major limb injury affecting treatment or rehabilitation protocol. Other than the demographic details, information concerning the duration of hospital stay, operating time (defined as the time from the skin incision to skin closure) and duration of leave were collected. All the cases included in this study were divided into two groups: patients treated with narrow 4.5-mm LCDCPs (group A) and those treated with 4.5-mm LCPs (group B). The average age in group A was 36.8 ± 8.9 years (range 18–65 years) and in group B was 37.6 ± 10.8 years (range 22–64 years). Both groups showed no statistical difference in term of age (*p* = 0.84), gender (*p* = 0.42), the time from injury to operation (*p* = 0.62), affected side (*p* = 0.58). There was no significant difference found between both the groups regarding distribution of fracture types (*p* value was 0.72, 0.68 and 0.42 for fracture types 12A, 12B and 12C, respectively). Demographic profiles of the two groups are shown in Table 1.

**Table 1:** Demographic profile of study

Characteristics	Group A	Group B
Age in years, Mean (range)	36.8 ± 8.9 years (18–65 years)	37.6 ± 10.8 years (22–64 years)
Sex (male:female)	73 : 29	75 : 35
Right:left	70 : 32	65 : 45
Preoperative radial nerve injury	8	10
Mechanism of injury		
Road traffic accident	51	46
Fall	26	29
Others	25	35
Fracture type(AO classification)		
Type 12 A	52	55
A1	25	27
A2	18	15
A3	9	13
Type 12 B	40	43
B1	14	16
B2	12	11
B3	14	16
Type 12 C	10	12
C1	4	6
C2	4	4
C3	2	2

**Fig. 1:**
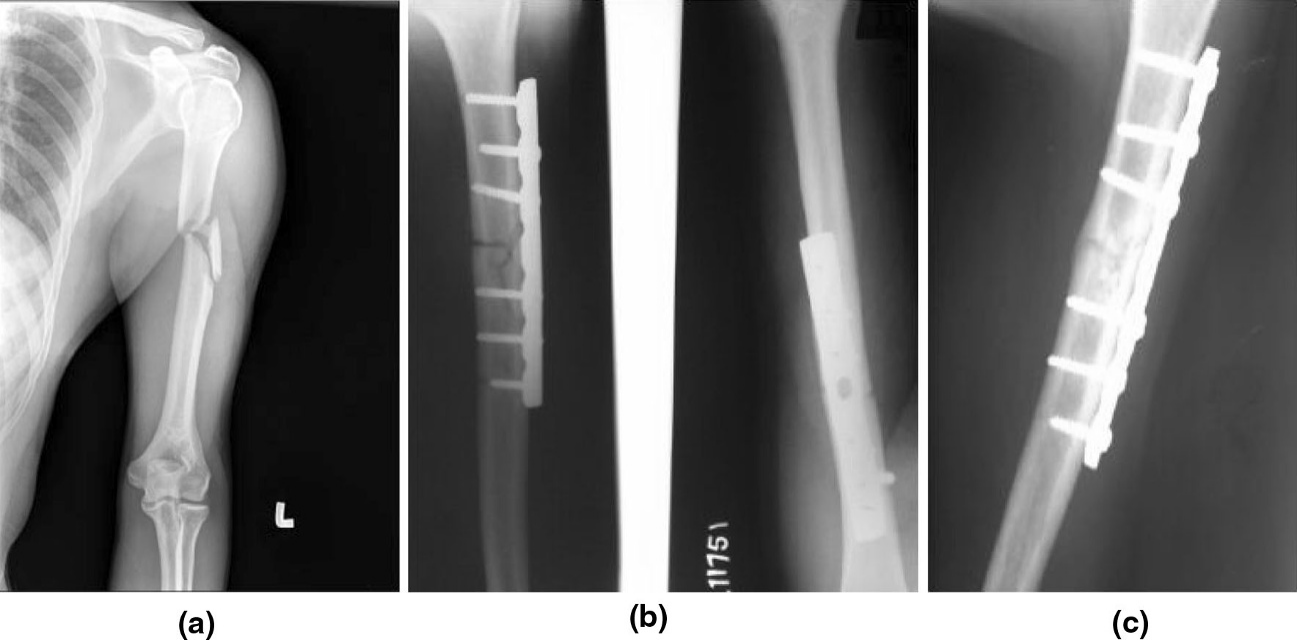
**a** Preoperative X-ray of 30-year-old male patient showing displaced fracture of the shaft of the humerus, left side. **b** Immediate postoperative X-ray anteroposterior (AP) and lateral view showing plate osteosynthesis with 4.5-mm narrow LCDCP. **c** Postoperative X-ray at 12-week follow-up visit showing a well-uniting fracture

**Fig. 2:**
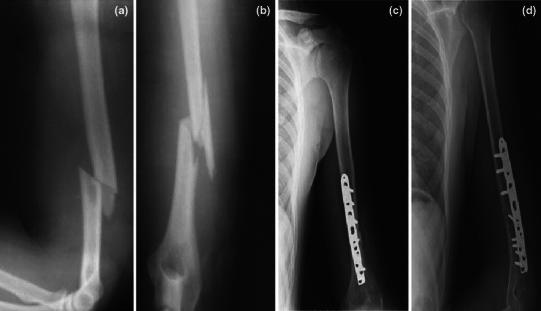
**a** and **b** Preoperative X-ray AP and lateral view showing displaced fracture of the shaft of the humerus, left side, in a 26-year-old female patient. **c** Immediate postoperative X-ray showing plate osteosynthesis with 4.5-mm LCP. **d** Postoperative X-ray at 12-week follow-up visit showing a well-uniting fracture

In all cases selected, patients were operated on between the 7th and 10th day after injury (range 2–21 days, average 8.0 days). All of the operations were performed under regional anesthesia, with the patient placed in the lateral decubitus position, using the posterior approach. The radial nerve was exposed and protected, then the fracture site was dissected to remove hematoma and soft tissue interposing between the fragments. The fracture fragments were reduced and plate osteosynthesis was done with either a 4.5-mm narrow LCDCP (group A) or an LCP (group B), using at least three screws in each end of the plate. Implants made by the same manufacturer (Synthes) were used in all the patients (Fig. 1a, b, c). Bicortical locking head screws were used in group B (Fig. 2a, b, c, d). The wound was closed after placing a suction drainage tube.

Patients were immobilized using a sling, while active and active-assisted range of motion began as soon as could be tolerated by the patient after surgery, generally on the 3rd day. All patients were followed up at 1 month intervals for the first 6 months after the surgery, and then at 2-month intervals for the next 6 months after surgery. Anteroposterior (AP) and lateral radiographs were taken at each follow-up visit. Shoulder and elbow range of motion was assessed at each follow-up visit.

Fracture union time, complications and functional outcomes were also recorded. The UCLA scoring system was used to assess shoulder function [10] and the Mayo elbow performance index (MEPI) [11] was used to assess elbow function. All patients were also evaluated on the basis of the outcome criteria of Rodriguez-Merchan [12] which consists of scores of shoulder and elbow movements along with pain and disability in the postoperative period, and has four categories of excellent, good, fair and poor outcomes.

The complications were evaluated in terms of infections (superficial or deep or chronic osteomyelitis), delayed union, nonunion, implant failure, secondary loss of reduction, implant breakage and refracture after plate removal. Malunion was defined as healing occurring at more than 15° of angulation. A delayed union was diagnosed when no satisfactory signs of healing were present at the 16-week follow-up visit. A nonunion was diagnosed when healing had not occurred after 6 months. Fractures which healed in <6 months were classified as unions.

Student’s *t*-test was used to analyze the difference of means for different parameters. The test was referenced for a two-tailed *p* value and a 95 % confidence interval was constructed around sensitivity proportion using normal approximation method. Statistical analyses were performed using SPSS software. A value of <0.05 was considered statistically significant.

## Results

The mean duration of injury for group A was 6.8 ± 2.8 days (range 2–10 days), while for group B it was 7.2 ± 3.2 days (range 1–14 days). There was no statistical significance between the two groups (*p* = 0.62). The mean operation time was 90.4 ± 40.6 min (range 70–140 min) in group A and 105.8 ± 30.1 min (range 68–150 min) in group B (*p* = 0.18). The incidence of iatrogenic radial nerve palsy in group B, 3.63 %, was insignificantly higher than in group A, 2.94 % (*p* = 0.52). The mean time to fracture union was 17.2 ± 6.8 weeks (range 10–48 weeks) in group A and 15.8 ± 5.1 weeks (range 12–42 weeks) in group B and there was no statistically significant difference found between the two groups (*p* = 0.28).

According to Rodriguez-Merchan criteria, at the 12-month follow-up visit, there was no significant difference regarding functional outcome of both the groups (*p* = 0.48) (Table 2). There was insignificant difference in the range of motion (ROM) and MEPI scores between the two groups. The mean ROM in group A was 130.82° ± 8.12° (range 110°–140°) and in group B was 134.68° ± 6.20° (range 120°–140°) (*p* = 0.28). The mean MEPI in group A was 98.98 ± 1.90 (range 92–100) and in group B was 99.68 ± 1.40 (range 94–100) (*p* = 0.54). At the 12-month follow-up visit, mean UCLA score in group A was 34.18 ± 0.62 (range 32–35) and 34.42 ± 0.42 (range 31–35) in group B, and there was no significant difference found between the two groups (*p* = 0.34).

**Table 2:** Comparison of functional outcomes of both groups

Group	Excellent	Good	Fair	Poor
LCDCP (group A)	73 (71.56 %)	19 (18.62 %)	8 (7.84 %)	2 (1.96 %)
LCP (group B)	83 (75.45 %)	17 (15.45 %)	7 (6.36 %)	3 (2.72 %)

**Fig. 3:**
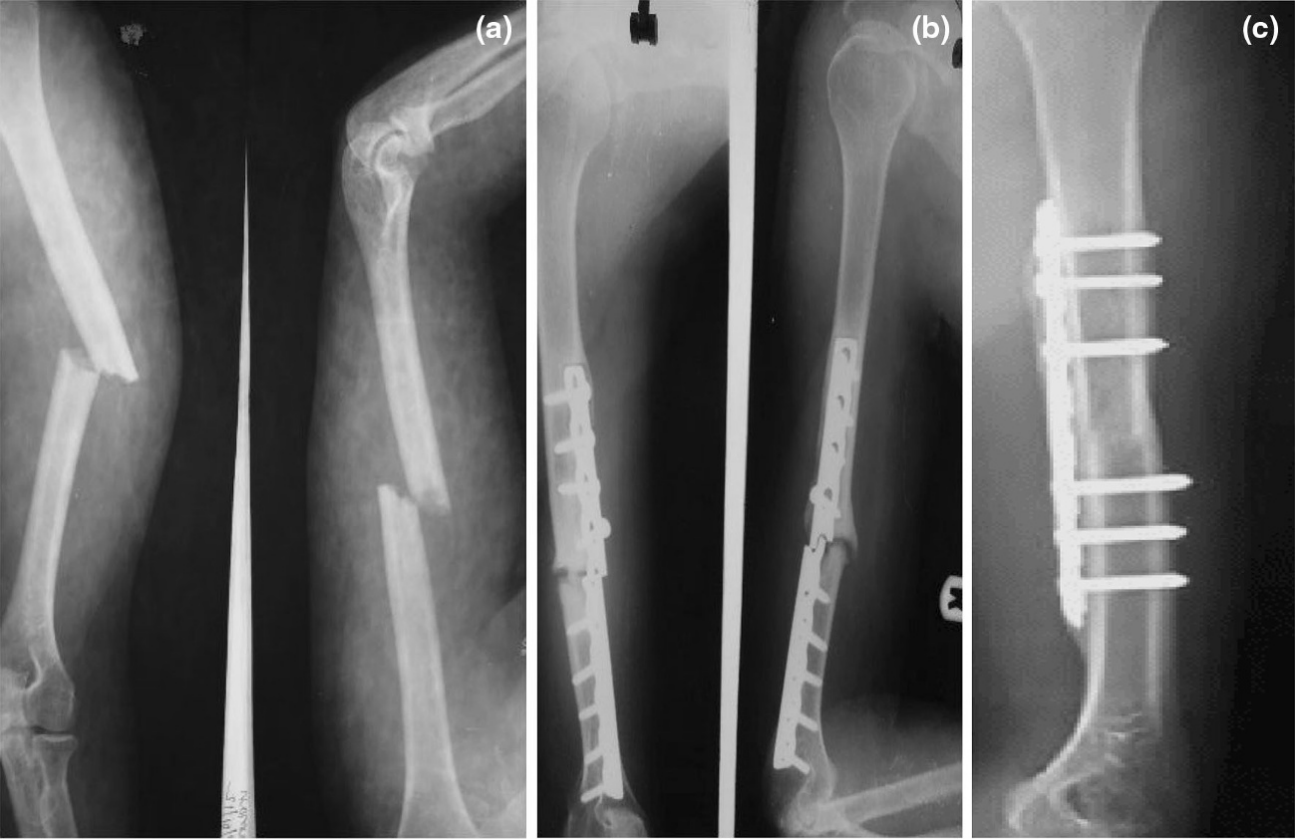
**a** Preoperative X-ray of a 45-year-old male patient showing a displaced fracture of the shaft of the humerus, right side. **b** Postoperative X-ray at 6-month follow-up showing implant (LCDCP) failure and nonunion at fracture sites. **c** Revision surgery (implant removal, freshening of fracture edges, internal fixation with 4.5-mm LCP) resulted in uneventful union of fracture site

Eight patients (7.84 %) in group A and seven patients (6.36 %) in group B had superficial infections (statistically insignificant difference, *p* = 0.68), which subsided uneventfully following antibiotic therapy. There was no incidence of deep infection in either group. All cases of preoperative radial nerve palsy in both groups recovered completely following stabilization. The radial nerve was explored in all these cases to check its integrity which was found to be intact in all the cases, indicating a neuropraxia type of injury. Three patients (2.94 %) in group A and four patients (3.63 %) in group B developed iatrogenic radial nerve palsies in the postoperative period, but there was no statistically significant difference found (*p* = 0.52) (Table 3). All seven cases of postoperative iatrogenic radial nerve palsies spontaneously recovered with conservative treatment with mean onset time of 18.6 weeks (range 10–42 weeks). Six cases in group A (5.88 %) and eight cases in group B (8.18 %) developed delayed union (Fig. 3a, b, c). All the patients were treated nonoperatively and had fracture union at 10.6 months (range 9–12 months) after the operation (Fig. 3c, d). Two patients in group A (1.96 %) and three in group B (2.72 %) had nonunion of fracture (insignificant difference, *p* = 0.24). Both cases of nonunion in group A also had implant failure while there was no incidence of implant failure in group B. There was no significant difference found (*p* = 0.07) between both the groups in terms of implant failure. Both cases in group A who had implant failure and nonunion of fracture were treated by revision surgery (implant removal, freshening of fracture edges, fixation with LCP and cancellous bone grafting) and they achieved uneventful union of the fracture site after revision surgery (Fig. 3a, b, c). All three cases of nonunion in group B were treated with cancellous bone grafting and they achieved union uneventfully. None of the cases in either group needed implant removal before the 12-month follow-up visit.

**Table 3:** Comparison of complications of both groups

Complications	Group A	Group B	*P* value
Infection	8 (7.84 %)	7 (6.36 %)	0.68
Iatrogenic radial nerve palsy	3 (2.94 %)	4 (3.63 %)	0.52
Delayed union	6 (5.88 %)	9 (8.18 %)	0.08
Nonunion	2 (1.96 %)	3 (2.72 %)	0.24
Implant failure	2 (1.96 %)	0	0.12
Refracture after implant removal	0	0	–

## Discussion

The internal fixation methods for humerus shaft fractures can be broadly grouped into plating or intramedullary techniques. Plate osteosynthesis remains the gold standard of fixation of humeral shaft fractures compared to other methods [13]. The reliability of union, together with early mobilization and return of the arm to normal function, favors the use of primary plate fixation in treatment of humeral diaphyseal fractures.

Shen et al. [14] retrospectively analyzed data from 43 patients with fractured humerus shafts treated with DCP and LCP using minimally invasive plate osteosynthesis (MIPO) techniques, and showed that there was no significant difference when outcomes and complications of the two types of implants were compared. Hur et al. [15] retrospectively analyzed data from 19 elderly patients with fractured humerus shafts treated with LCDCP and LCP. In their study, loosening of the plate occurred in one case each from the LCP group and the LCDCP group. The rest of the patients achieved union uneventfully without any complications. Union rate and clinical scores were not significantly different between the two groups. They advised that the principle of fracture fixation was more important than plate selection in humeral shaft fractures of elderly patients. Results of the present study are comparable with the reported literature [14, 15]. In their prospective study, Sommer et al. [16] published the results of use of various LCPs in treatment of 144 patients with 169 fractures, and concluded that the LCP was a technically mature option in complex fracture situations and in revision operations after the failure of other implants. Ring et al. [17] treated 24 patients with osteoporotic delayed union (9 patients) and nonunion (15 patients) of the humeral diaphysis with LCP. All the fractures eventually healed and, using a modification of the Constant and Murley shoulder score, the results were good or excellent in 22 patients and fair in 2 patients.

Gardener et al. [18] compared the mechanical behavior under cyclic loading of LCP constructs and LCDCP constructs. Traditional compression plating failed significantly earlier in torsion. In AP bending, traditional constructs demonstrated significantly greater energy absorption, suggesting greater deformation. Fracture motion and stiffness measurements were discordant in the LCDCP specimens in torsion. In contrast, the LCP specimen had no discordance in stiffness and fracture motion. On the other hand, many of the other parameters compared between the two plates showed no difference, and the overall clinical advantage of locked plates is subtle. Xiong et al. [6] also showed in their cadaveric study that the LCP has a lower interface contact area and lower average force than that of the LCDCP and that the LCP is a good alternative for treating forearm and humerus diaphyseal fractures. In their study, Hoerdemann et al. [5] compared the in vitro biomechanical characteristics of LCDCP and LCP constructs in an osteotomy gap model of femoral fracture in neonatal calves and showed that insertion torque sufficient to provide adequate stability in femurs of newborn calves could not be achieved reliably with 4.5-mm cortical screws, and that LCP constructs were significantly more resistant to compression than LCDCP constructs. Leung and Chow [19] compared LCDCP with PC-Fix and LCP in treatment of closed forearm fractures in their randomized control trial and said that the LCP is effective for use as a bridging device in treating comminuted fractures; its usage in simple fractures and its superiority over conventional plating systems is yet to be proved.

The limitation of our study was small sample size in both groups and absence of long-term follow-up. A randomized control trial, preferably triple blinded or at least double blinded in nature, involving a large number of patients with long-term follow-up is needed to evaluate significant differences between LCDCP and LCP fixation in fractures of the shaft of the humerus.

Our study concludes that the final outcome is determined by using proper principles of plating and it is the proper application of the principles of plating and not the type of plate which decides outcomes and complications.
